# Thermal physiology and activity in relation to reproductive status and sex in a free-ranging semelparous marsupial

**DOI:** 10.1093/conphys/coz073

**Published:** 2019-11-11

**Authors:** Cassandra A Parker, Fritz Geiser, Clare Stawski

**Affiliations:** 1 Centre for Behavioural and Physiological Ecology, Zoology, University of New England, Armidale, NSW 2351, Australia; 2 Department of Biology, Norwegian University of Science and Technology, Trondheim 7491, Norway

**Keywords:** body temperature, lactation, mammal, pregnancy, torpor

## Abstract

In a changing climate, southern hemisphere mammals are predicted to face rising temperatures and aridity, resulting in food and water shortages, which may further challenge already constrained energetic demands. Especially semelparous mammals may be threatened because survival of the entire population depends on the success of a single breeding event. One of these species, the yellow-footed antechinus, *Antechinus flavipes*, a small, heterothermic marsupial mammal, commences reproduction during winter, when insect prey is limited and energetic constraints are high. We examined the inter-relations between thermal and foraging biology of free-ranging *A. flavipes* and examined whether they use torpor for energy conservation, despite the fact that reproduction and torpor are considered to be incompatible for many mammals. Females used torpor during the reproductive season, but patterns changed with reproductive status. Prior to breeding, females used frequent (86% of days), deep and long torpor that was more pronounced than any other reproductive group, including pre-mating males (64% of days). Pregnant females continued to use torpor, albeit torpor was less frequent (28% of days) and significantly shorter and shallower than before breeding. Parturient and lactating females did not express torpor. During the mating period, males reduced torpor use (24% of days). Pre-reproductive females and pre-mating males were the least active and may use torpor to minimize predator exposure and enhance fat deposition in anticipation of the energetic demands associated with impending mating, gestation and lactation. Reproductive females were most active and likely foraged and fed to promote growth and development of young. Our data show that *A. flavipes* are balancing energetic demands during the reproductive season by modifying torpor and activity patterns. As the timing of reproduction is fixed for this genus, it is probable that climate change will render these behavioural and physiological adaptations as inadequate and threaten this and other semelparous species.

## Introduction

Australia has a poor record of mammal extinctions, with >10% of the continent’s endemic land mammal species believed to have become extinct during the past ~200 years (Woinarski *et al*., 2015). Extinctions have been taxonomically uneven, with native rodents and marsupial macropods and bandicoots being most affected (Johnson, 2006; Woinarski *et al*., 2015). Although to date carnivorous marsupials (family Dasyuridae) have suffered range reductions, otherwise they have fared rather well, but the unusual reproductive pattern of some makes them potentially vulnerable to climate change. Many small dasyurids of the genera *Antechinus*, *Phascogale* and *Dasykaluta* are semelparous (death after first reproduction) with a single, photoperiod-dependent and temporally fixed reproductive event per year in late winter, often followed by complete male die-off (Woolley, 1966; McAllan and Dickman, 1986; Bradley, 2003; Fisher *et al*., 2013). Females typically perish shortly after their young have been weaned in late summer (Woolley, 1966). This unusual mode of reproduction with a single cohort of pregnant and lactating females and no adult males is risky as it depends on survival of these females. This in turn depends on adequate synchronization of the reproductive event and a reliable spring flush of insects to ensure that food is plentiful during lactation, the energetically most demanding reproductive state.

Marsupials have a slow developmental rate (Lee and Cockburn, 1985). Consequently, to ensure that lactation occurs in spring, mating must occur in winter when energy costs for thermoregulation are high because of the low ambient temperatures (*T*_a_) and insect availability is generally low (Withers *et al*., 2016). Many non-reproductive mammals use torpor, a controlled reduction of metabolism and body temperature (*T*_b_), to deal with such conditions for energy conservation (Boyer and Barnes, 1999; Ruf and Geiser, 2015). Torpor is potentially important in the context of Australian mammalian extinctions, because heterothermic species that express torpor have a reduced extinction rate in comparison to homeothermic species that cannot use torpor (Geiser and Turbill, 2009; Hanna and Cardillo, 2014). However, during reproduction torpor is avoided by some but not all heterothermic mammals (McAllan and Geiser, 2014). Torpor use by reproductive mammals has been observed for a number of taxa including monotremes, bats, mouse lemurs and, relevant to our work, also some dasyurid marsupials (Willis *et al*., 2006; Morrow and Nicol, 2009; Stawski, 2010; Dzal and Brigham, 2013; Klug and Barclay, 2013; McAllan and Geiser, 2014). Importantly, heterothermy and the effective use of torpor also enhance fat storage, which can be used later when energetic demands increase, such as during lactation, increasing the chance of survival for mother and offspring (Geiser and Masters, 1994).

To unravel the importance of balancing energy expenditure (activity) with energy savings (torpor), we studied a semelparous dasyurid marsupial the yellow-footed antechinus, *Antechinus flavipes*, a small (~30 g), heterothermic mammal endemic to Australia. *Antechinus flavipes* commence reproduction during late winter when insect prey is limited and energetic constraints are high. The breeding period for *A. flavipes* is highly synchronized, lasting no more than 2 weeks, and occurring at the same time year to year at any given locality (McAllan and Dickman, 1986; Naylor *et al*., 2008), although there is some variation among populations across latitudes (Smith, 1984). The environmental cue that appears to stimulate the onset of sexual activity is the rate of change in photoperiod (McAllan and Geiser, 2006), which will not be affected by climate change. *Antechinus flavipes*, as other related marsupials, currently occupy predictable environments where optimal conditions for lactation and weaning occur punctually each year during spring coinciding with an associated rise in temperature and insect numbers.

As photoperiod is predictable and constant, whereas climate change will shift seasonal food availability, animals that are reliant upon reproductive events that occur in response to changes in photoperiod may be negatively affected by climate change. Therefore, we examined the thermal and foraging biology of free-ranging male and female *A. flavipes*, and specifically the inter-relations between activity and expression of torpor, throughout the reproductive period including male die-off. The use of daily torpor is well documented for non-reproductive antechinus, both for captive and for free-ranging individuals (Geiser, 1988; Rojas *et al*., 2014). However, the role of torpor as a strategy for reproductive success, and therefore population persistence in small free-ranging semelparous marsupials, is poorly understood yet is likely to play a profound role with regard to extinction risk in this group. Data on thermal biology for reproductive female antechinus are restricted to two captive individuals (Stawski and Rojas, 2016), which typically express less torpor than in the wild (Geiser *et al*., 2000). Based on these captive data, we hypothesized that (*i*) daily body temperature (*T*_b_) fluctuations will vary throughout the reproductive period, with more torpor being employed early during the reproductive period due to colder temperatures and a decreased food supply; (*ii*) females will reduce torpor use during pregnancy and lactation; and (*iii*) males will employ less torpor and are more active than females as they need to seek mating opportunities.

## Material and methods

Our study was conducted at Aberbaldie Nature Reserve, in New South Wales, Australia (31°04′24″ S, 151°25′34″ E). Trapping of *A. flavipes* was conducted during late June through to early July 2016 (austral mid-winter), before breeding commenced. Individuals were captured using Elliott aluminium box traps (Elliott Scientific Equipment, Upwey, Australia). Climatic conditions were inclement during the trapping period (rain and snow); therefore, Dacron polyester fibre was provided for insulation inside the traps, and a small plastic bag was fitted over the closed end of each trap to keep the interior dry. A total of 15 individuals (6 males, 9 females) were captured and implanted with transmitters (see below).

A second trapping effort was conducted during September (austral Spring) to confirm whether parturition had occurred in the remaining females, verifying interpretations of *T*_b_ fluctuations in relation to reproductive stage. This second trapping effort was concluded following the confirmation of pouch young for the remaining study females.

The *T*_a_ in the reserve was measured at 10-min intervals using iButtons (± 0.5°C, iButton DS1921G, Maxim Integrated Products, Sunnyvale, CA, USA) placed in an inverted Styrofoam cup in the shade ~2 m above the ground.

To record the *T*_b_ and activity patterns of the study animals, we used temperature-sensitive radio transmitters (1.9–2.5 g, Sirtrack, Havelock North, New Zealand), which were calibrated to the nearest 0.1°C prior to implantation with a precision mercury thermometer traceable to a national standard. The pulse interval of each transmitter, with an individual transmission frequency for each animal, was recorded in 5°C increments for temperatures between 15 and 45°C. As pulse interval is a function of temperature, transmitter-specific calibration curves (*R*^2^ ≥ 0.99) were then produced for each transmitter. Each transmitter was coated with inert wax (Paraffin/Elvax) before implantation.

Animals were anaesthetized using gaseous isoflurane/oxygen, before a small incision was made to the abdomen for insertion of a transmitter weighing <8% of the individual’s body mass (BM) (Rojas *et al*., 2010) into the intraperitoneal cavity. The incision was closed (muscle and skin were sutured separately) using coated Vicryl (3.0 metric, Ethicon Inc.). A topical anaesthetic (Xylocaine, AstraZeneca Pty Ltd, North Ryde, NSW, Australia) and Leuko Spray Bandage (BSN medical (Aust) Pty Ltd, Clayton, VIC, Australia) were applied to the surgical site. The animals were provided with food and water *ad libitum* before being released at the site of capture. All procedures were approved by the University of New England Animal Ethics Committee and the New South Wales National Parks and Wildlife Service.

Radio-tracking of individual *A. flavipes* was conducted from July to October, although some individuals died or became undetectable before the end of the study, most likely due to die-off, predation or battery failure. Individuals were radio-tracked to their nest site every morning from 1 July until 14 September and then every 3 days until 13 October using a hand-held receiver (Icom IC-R10, Osaka, Japan) and Yagi antenna (Titley Scientific, Australia). A mobile receiver/logger system consisting of a receiver and data logger and connected to a H-frame antenna (Titley Electronics, model AH/C, Ballina, NSW, Australia) (see Körtner and Geiser, 1998) was deployed at the nest site of each animal. The receiver/logger recorded the pulse interval of the selected transmitter every 10 min. Data were downloaded periodically and converted to *T*_b_ using the individual calibration curves. Due to a short detection range, receiver/loggers were required to be relocated whenever an individual moved to a new nest site.

*A. flavipes* were considered to be torpid when *T*_b_ fell below 31.5°C. The torpor threshold was determined by applying mean BM and mean *T*_a_ to the following equation (Willis 2007): *T*_b-onset_—1 SE = (0.041) BM + (0.040) *T*_a_ + 31.083.

For the current study, mean BM for males was 29.5 ± 2.4 g (*n* = 6), and that of females 25.2 ± 4.9 g (*n* = 9), and mean *T*_a_ during the course of the study was 7.5 ± 3.0°C. The above equation has been used in a number of recent studies of torpor use in antechinus (e.g. Stawski and Rojas, 2016; Matthews *et al*., 2017) as it allows shallow, yet energetically significant, torpor bouts to be recognized. However, torpor thresholds between *T*_b_ 31 and 32°C are often also used for heterothermic rodents and other mammals (Barclay *et al*., 2001).

Torpor entry and arousal times and torpor bout duration (TBD) were determined from periods when *T*_b_ was below the torpor threshold for ≥30 min (Geiser and Masters, 1994). For analysis, we combined all torpor bouts for a single day and termed it total daily torpor (TDT). Departure and arrival times from the nest, and duration of activity, were determined from the time an animal was absent from the range of the logger, until it was within range again. Torpor bouts were excluded if large gaps existed in the data, and activity bouts were excluded if the animal moved to a new nest, as the return time could not be determined.

Pregnancy for females was calculated as occurring 31.5 days before the first day of the parturition period (Selwood and Woolley, 1991; McAllan and Geiser, 2006), which was characterised by a 5-day period of strict homeothermy (Stawski and Rojas, 2016). Female reproductive condition was categorized as one of four groups: pre-reproductive (females prior to breeding; *n* = 9), pregnant (females in the 31.5-day period prior to parturition; *n* = 5), parturient (5-day period of strict homeothermy during the time of birth; *n* = 4) and lactating (milk production/young attached to teats; *n* = 3). Seven of the females studied were first-year females and two were second-year females. However, as no significant differences were found between these groups for any of the variables, they were analysed together. Males were placed in two categories: pre-mating (*n* = 6) and mating (*n* = 3).

The data analyses were conducted using ‘R’ Studio (R Development Core Team, 2009), following tests for normality (*Q*–*Q* plot) and homoscedasticity (residual plot). Linear mixed-effects (lme) models were then fitted (package ‘nlme’) to determine whether reproductive condition had an effect on the measured variables (TDT, daily mean *T*_b_ (*T*_b mean_), daily minimum *T*_b_ (*T*_b min_), daily range *T*_b_ (*T*_b range_) and total activity duration (TAD)). In addition, daily minimum *T*_a_ (*T*_a min_) and initial BM were included as covariates for all variables. To account for repeated measures, individuals were included as a random effect. If there was a significant effect, a post hoc Tukey test (package ‘multcomp’) was performed to determine which reproductive groups differed. *R*^2^ values for regression models are provided (packages ‘lme4’ and ‘MuMIn’). To compare the proportions of days that torpor was used across reproductive groups, a two-factor logistic regression with a binomial error structure was used (function ‘aov’ and package ‘multcomp’). The number of individuals (*n*) and the number of observations (*N*) are provided where applicable. Means were derived by averaging the mean of all individuals in each group and are presented with ±1 standard deviation (SD). A significance level of *P* < 0.05 was used throughout the study.

## Results

### Weather variables

Throughout the study period, daily mean *T*_a_ was 7.5 ± 3.0°C (*N* = 122; range: −0.3 to 18.0°C). Daily mean minimum *T*_a_ was 3.5 ± 3.0°C (*N* = 122; range: −3.0 to 11.5°C), and daily mean maximum *T*_a_ was 13.0 ± 4.5°C (*N* = 122; range: 3.0 to 26.5°C). The daily mean range of *T*_a_ was 9.5 ± 4.0°C (*N* = 122; range: 2.5 to 17.5°C).

### Study animals and survivorship

A total of *n* = 6 males and *n* = 9 females were captured and implanted with transmitters. Mean BM at the time of capture for males was 29.5 ± 2.4 g, and that for females was 25.2 ± 4.9 g; this did not differ significantly between males and females (*F*_1,13_ = 3.86, *P* = 0.071). During the course of the study, two males and four females died and their body and/or transmitter was retrieved. A further four males and one female went missing before the conclusion of the study, likely a consequence of either transmitter failure, die-off or predation.

Nest sites were predominantly large (>150 cm circumference) dead, hollow trees, many of which were burnt and open-topped. Some individuals occasionally nested in rock piles or crevices. Nest trees were often shared between multiple study animals, with one nest tree housing up to six individuals with transmitters on a number of occasions. We do not know if there were additional individuals in these nests without transmitters. The sex ratio of tagged individuals in these shared nests was often equal. Individuals that regularly shared nests appeared to form a stable group, and although they separated on occasion when changing nests, they typically re-joined the group after a few days.

All males were either dead or undetectable by 6 September. Of the two male carcasses that were found, one had clearly been killed by a predator as only the transmitter remained, and, although the other male was intact, wounds to the body suggested it had also been attacked by a predator. This individual also had a number of external parasites. Both of these males died after the breeding period had commenced.

All nine females were alive throughout the mating period. However, three were found deceased in hollow logs at the conclusion of the mating period (recovered between 20 August and 5 September), while a fourth female appeared to have been predated upon as only the transmitter could be recovered (also following the conclusion of the mating period). A fifth female was undetectable after 1 September, presumably either because of transmitter failure or predation. Four surviving female *A. flavipes* were detectable during the period of parturition, which ranged from 14 to 28 September. Conception in these females therefore occurred between 14 and 23 August.

### Torpor

**Figure 1:**
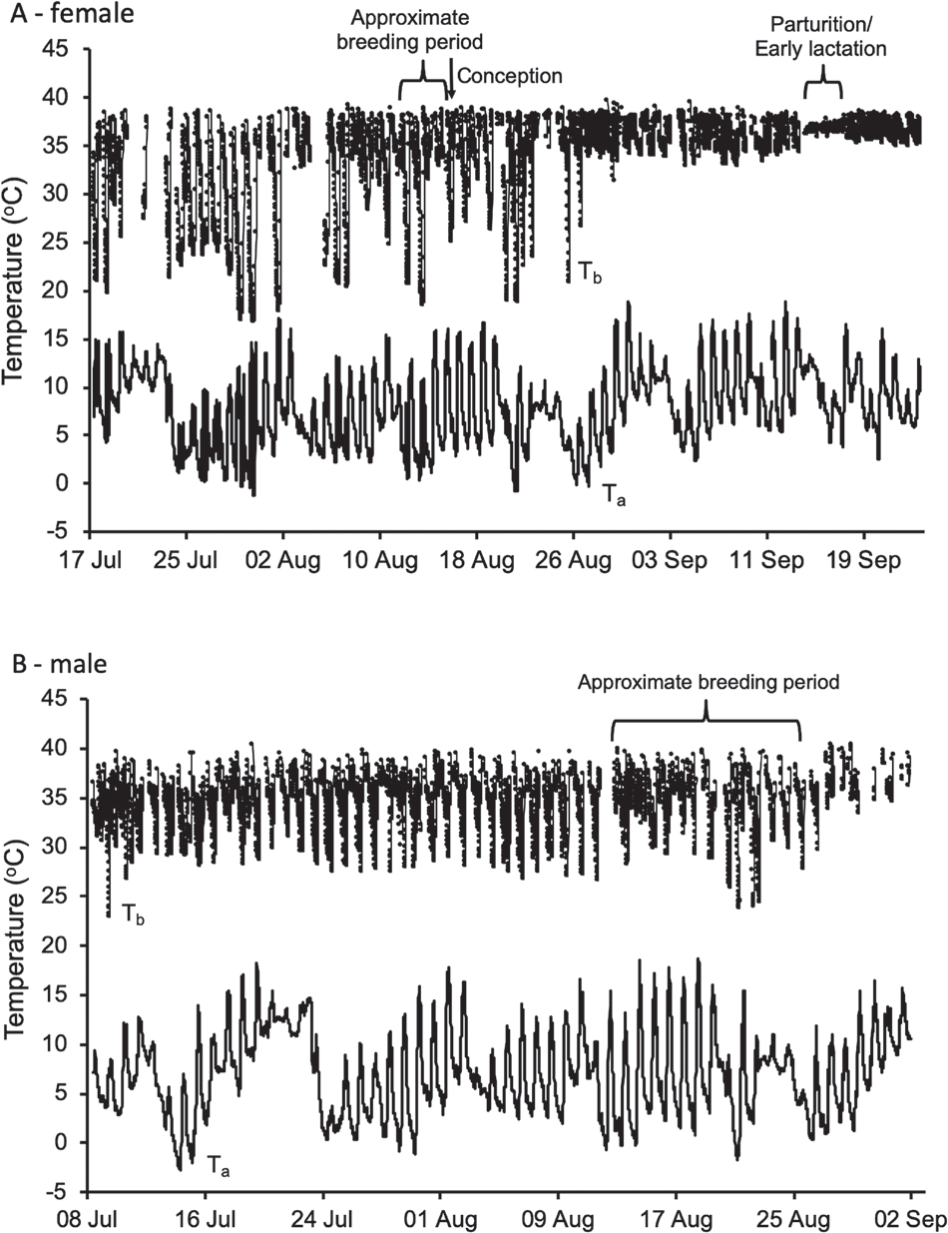
(**A**) Body temperature (*T*_b_; dots) fluctuations of a female *Antechinus flavipes* and ambient temperature (*T*_a_; line) throughout the study period. Also shown are the approximate breeding period, the date of conception and parturition/early lactation. (**B**) *T*_b_ fluctuations of a male *A. flavipes* throughout the study period including *T*_a_. The approximate breeding period is also shown.

Female *A. flavipes* expressed at least three distinct patterns of torpor use, which largely coincided with changes in reproductive condition (Fig. 1A). Male *A. flavipes* decreased torpor use during the mating period (Fig. 1B).

**Figure 2:**
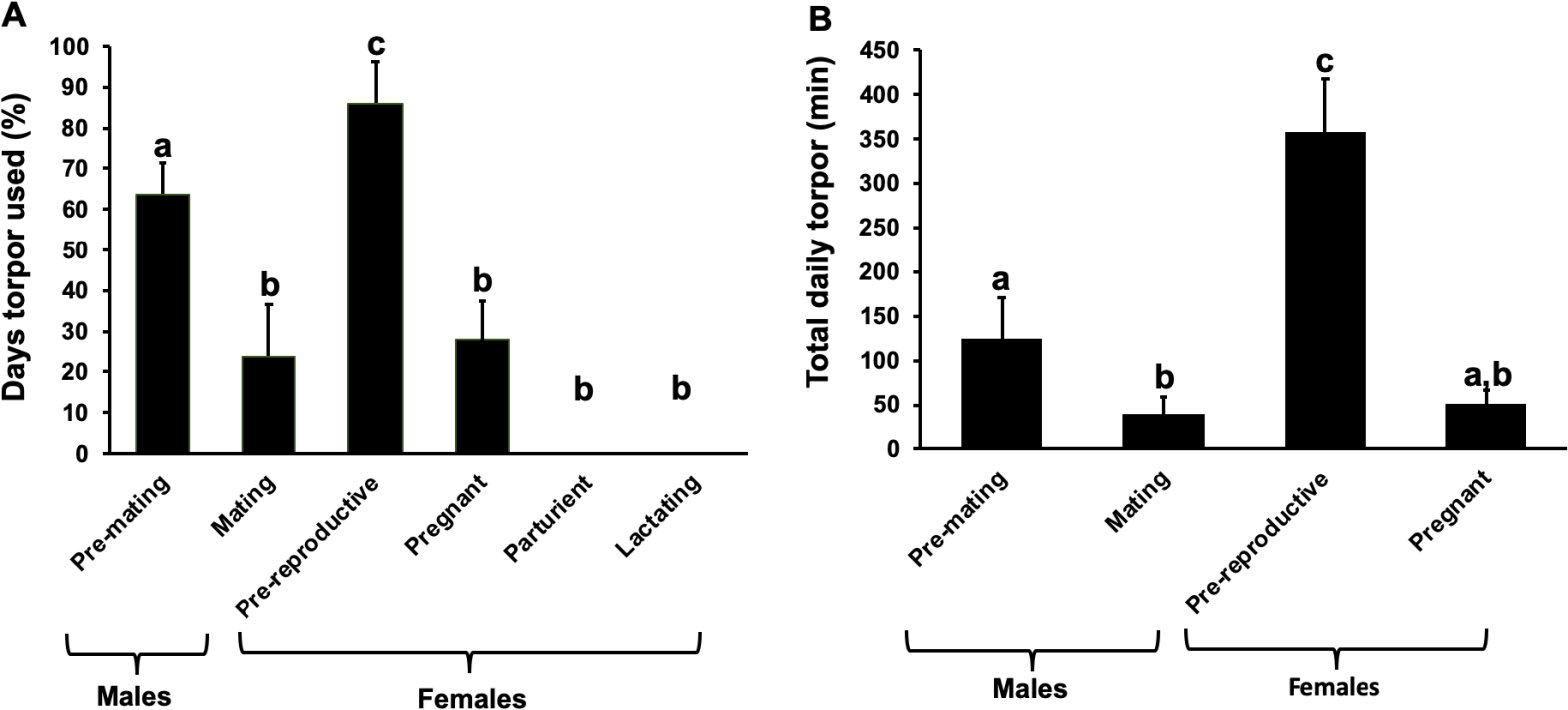
(**A**) Mean proportion of days that torpor was used (percent) by individuals and (**B**) mean TDT (min) is compared in individuals of varying reproductive condition. Parturient and lactating females were excluded from TDT analysis because they did not use any torpor. Significantly different groups are marked with different letters (post hoc Tukey test)

The reproductive group had a significant effect on the proportion of days that individuals employed torpor (*F*_5,24_ = 49.3, *P* < 0.0001, two-factor logistic regression with a binomial error structure). Pre-reproductive females (*n* = 9) entered torpor on 86.1 ± 10.0% of days, the highest proportion of torpor days of any reproductive group (*P* < 0.001 in comparison to all other reproductive groups, post hoc Tukey test), while pre-mating males (*n* = 6) entered torpor on 63.9 ± 7.5% of days (Fig. 2A). Males during the mating period reduced the times they entered torpor to 23.9 ± 12.6% of days (*n* = 3). Parturient (*n* = 4) and lactating (*n* = 3) females never entered torpor, whereas pregnant females (*n* = 5) did use some torpor, albeit infrequently (27.8 ± 9.7% of days). These three reproductive groups and males during the mating period were not significantly different in their proportion of torpor use days (*P* = 0.075 up to 1.000 between these groups, post hoc Tukey test; Fig. 2A).

Both reproductive group (*t*_13,721_ = 12.2, *P* < 0.0001, lme) and daily *T*_a min_ (*t*_13,721_ = −11.0, *P* < 0.0001, lme) significantly influenced mean TDT. Mean TDT of pre-reproductive females was 358.2 ± 58.9 min (*n* = 8, *N* = 400), significantly longer than all of the other reproductive groups using torpor (pregnant females: 51.8 ± 14.6 min, *n* = 5, *N* = 188, *P* < 0.0001, *z* = 12.15, post hoc Tukey test; pre-mating males: 125.1 ± 46.2 min, *n* = 6, *N* = 179, *P* < 0.0001, *z* = 9.02, post hoc Tukey test; mating males: 40.3 ± 18.7 min, *n* = 3, *N* = 43, *P* < 0.0001, *z* = 8.29, post hoc Tukey test; Fig. 2B). The difference in TDT between pregnant females and pre-mating males (*P* = 0.771, *z* = 0.94, post hoc Tukey test) and mating males (*P* = 0.354, *z* = −1.61, post hoc Tukey test) was not significant (Fig. 2B). However, TDT was significantly lower in mating males in comparison to pre-mating males (*P* = 0.012, *z* = −3.03, post hoc Tukey test). The longest TDT during a single day for a pre-reproductive female was 1160 min, while for pregnant females the recorded maximum TDT was 550 min. For pre-mating males, maximum TDT was 670 min and that for mating males 260 min. Decreasing *T*_a_ was correlated with increasing TDT but was significant only for pre-reproductive females (*t*_7,390_ = −9.95, *P* < 0.0001, *R*^2^ = 0.23, *y* = −39.16*x* + 470.77, lme), pregnant females (*t*_3,112_ = −3.79, *P* < 0.0001, *R*^2^ = 0.12, *y* = −13.83*x* + 119.50, lme) and pre-mating males (*t*_4,171_ = −4.35, *P* < 0.0001, *R*^2^ = 0.16, *y* = −12.25*x* + 158.98, lme). TDT for mating males was not significantly influenced by *T*_a_ (*t*_1,40_ = −1.44, *P* = 0.158, lme).

**Figure 3:**
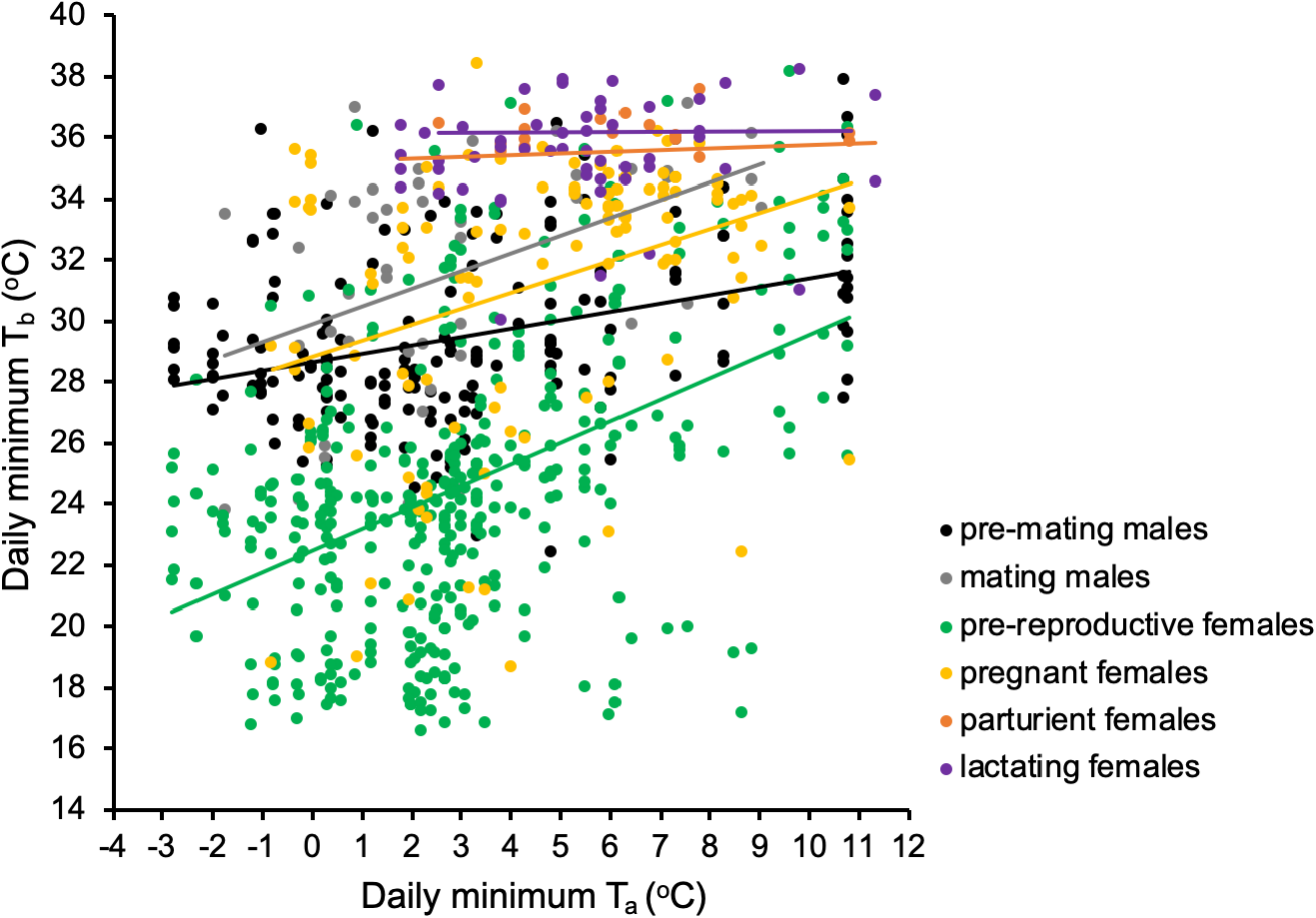
The relationship between daily minimum body temperature (*T*_b min_) and daily minimum ambient temperature (*T*_a min_) among reproductive groups. The relationship was significant in pre-mating males (*t*_4,171_ = 5.21, *P* < 0.0001, *R*^2^ = 0.26, *y* = 0.32*x* + 28.97), mating males (*t*_1,40_ = 3.19, *P* = 0.003, *R*^2^ = 0.26, *y* = 0.59*x* + 29.89), pre-reproductive females (*t*_7,390_ = 10.27, *P* < 0.0001, *R*^2^ = 0.22, *y* = 0.71*x* + 22.46) and pregnant females (*t*_3,112_ = 3.78, *P* = 0.003, *R*^2^ = 0.11, *y* = 0.53*x* + 28.84). *T*_a min_ had no obvious effect on *T*_b min_ in parturient females (*t*_2,12_ = 0.62, *P* = 0.546) or lactating females (*t*_1,49_ = 0.70, *P* = 0.486).

### Body temperature

Reproductive group (*t*_13,789_ = 3.8, *P* < 0.0001, lme) and daily *T*_a min_ (*t*_13,789_ = 10.8, *P* < 0.0001, lme) revealed a significant effect on daily *T*_b mean_. Daily *T*_b mean_ for pre-reproductive females was the lowest of all reproductive groups (31.9 ± 0.4, *n* = 9, *N* = 400; Table 1; *P* < 0.0001 in comparison to all other groups, post hoc Tukey test). *T*_b mean_ for pre-mating males (34.6 ± 0.5, *n* = 6, *N* = 178) and pregnant females (35.3 ± 0.5, *n* = 5, *N* = 118) did not differ significantly (Table 1; *P* = 0.994, *z* = −0.53, post hoc Tukey test), but were lower in comparison to mating males (35.7 ± 0.7, *n* = 3, *N* = 44) and parturient (37.1 ± 0.5, *n* = 5, *N* = 17) and lactating (36.5 ± 0.8, *n* = 3, *N* = 53) females (*P* < 0.04 for these comparisons, post hoc Tukey test). Mating males and parturient and lactating females did not differ in *T*_b mean_ (Table 1; *P* > 0.997 for these groups, post hoc Tukey test).

**Table 1:** Summary of body temperature (*T*_b_) data from *Antechinus flavipes*. If means differed significantly between the groups, they are identified by different letters

	**Males**		**Females**			
**Variable**	**Pre-mating**	**Mating**	**Pre-reproductive**	**Pregnant**	**Parturient**	**Lactating**
Absolute *T*_b_ min (°C)	22.4	23.8	16.6	18.7	35.3	32.2
Absolute *T*_b_ max (°C)	41.0	40.5	41.5	40.0	39.8	39.4
Mean *T*_b_ (°C)	34.6 ± 0.5^a^	35.7 ± 0.7^b^	31.9 ± 0.4^c^	35.3 ± 0.5^a^	37.1 ± 0.5^b^	36.5 ± 0.8^b^
Mean *T*_b_ min (°C)	29.7 ± 1.2^a^	31.3 ± 0.7^b^	24.6 ± 0.6^c^	31.3 ± 0.5^b^	36.2 ± 0.3^d^	35.3 ± 0.8^d^
Mean *T*_b_ max (°C)	39.1 ± 0.4^a^	39.3 ± 0.2^a,b^	38.6 ± 0.6^a,b,c^	38.5 ± 0.7^a,c,d^	37.9 ± 0.5^d^	38.3 ± 0.4^a,c,d^
Mean *T*_b_ range (°C)	9.4 ± 1.3^a^	7.7 ± 0.8^b^	14.0 ± 1.1^c^	7.8 ± 1.0^b^	1.8 ± 0.5^d^	3.0 ± 0.3^d^

Daily *T*_b min_ was significantly affected by both reproductive group (*t*_13,789_ = 6.3, *P* < 0.0001, lme) and daily *T*_a min_ (*t*_13,789_ = 12.1, *P* < 0.0001, lme). Pre-reproductive females expressed the lowest daily mean *T*_b min_ of any reproductive group (Table 1; *P* < 0.001 in comparison to all other groups, post hoc Tukey test). Pre-mating males displayed a significantly lower daily mean *T*_b min_ than mating males (Table 1; *P* = 0.006, *z* = 3.48, post hoc Tukey test), and pregnant females were similar to the mating males (Table 1; *P* = 0.381, *z* = 1.89, post hoc Tukey test). Parturient and lactating females maintained the highest mean *T*_b min_ of all reproductive groups (Table 1; did not differ from each other, *P* = 0.999, *z* = 0.27, post hoc Tukey test). Daily mean *T*_b min_ showed a linear relationship with *T*_a min_ where a lower daily *T*_a min_ generally led to a lower daily *T*_b min_. The relationship was strongest for pre-mating males (*t*_4,171_ = 5.21, *P* < 0.0001, *R*^2^ = 0.26, *y* = 0.32*x* + 28.97, lme) and mating males (*t*_1,40_ = 3.19, *P* = 0.003, *R*^2^ = 0.26, *y* = 0.59*x* + 29.89, lme), followed by pre-reproductive females, (*t*_7,390_ = 10.27, *P* < 0.0001, *R*^2^ = 0.22, *y* = 0.71*x* + 22.46, lme) and pregnant females (*t*_3,112_ = 3.78, *P* = 0.003, *R*^2^ = 0.11, *y* = 0.53*x* + 28.84, lme; Fig. 3). This correlation was not significant for lactating females (*t*_1,49_ = 0.70, *P* = 0.486, lme) or parturient females (*t*_2,12_ = 0.62, *P* = 0.546, lme; Fig. 3).

Both reproductive group (*t*_13,789_ = 2.2, *P* = 0.002, lme) and daily *T*_a min_ (*t*_13,789_ = 3.4, *P* = 0.0008, lme) displayed a significant effect on daily mean *T*_b max_. Daily mean *T*_b max_ was highest for mating males and was 1.3°C lower for parturient females (Table 1; *P* = 0.001, *z* = −3.79, post hoc Tukey test). Mating males also had a significantly higher *T*_b_ in comparison to pregnant (Table 1; *P* = 0.039, *z* = 2.82, post hoc Tukey test) and lactating (Table 1; *P* = 0.011, *z* = 3.23, post hoc Tukey test) females. Additionally, parturient females displayed a lower daily mean *T*_b max_ in comparison to pre-mating males (Table 1; *P* = 0.009, *z* = −3.29, post hoc Tukey test) and pre-reproductive females (Table 1; *P* = 0.030, *z* = −2.92, post hoc Tukey test). Daily mean *T*_b max_ did not differ significantly in any of the other reproductive groups (Table 1; *P* = 0.063 up to 0.925, post hoc Tukey test).

Daily fluctuations in *T*_b range_ were significantly influenced by the reproductive group (*t*_13,789_ = 12.8, *P* < 0.0001, lme) and *T*_a min_ (*t*_13,789_ = −11.1, *P* < 0.0001, lme). The absolute lowest and highest *T*_b_ values in the study were exhibited by pre-reproductive females (Table 1). Therefore, unsurprisingly, pre-reproductive females expressed the greatest fluctuations in *T*_b range_ (Table 1; *P* < 0.0001 in comparison to all other groups, post hoc Tukey test). *T*_b range_ was significantly lower for pre-mating males (Table 1; *P* < 0.0001, *z* = 7.7, post hoc Tukey test), but this group had larger daily *T*_b_ fluctuations than mating males (Table 1; *P* = 0.027, *z* = −3.00, post hoc Tukey test) and pregnant females (Table 1; *P* = 0.002, *z* = 3.73, post hoc Tukey test). The lowest *T*_b range_ was observed for parturient females (Table 1). Even though the mean *T*_b range_ for lactating females was 1.2°C greater (Table 1), the difference between parturient and lactating females was not significant (*P* = 0.984, *z* = −0.65, post hoc Tukey test).

### Activity

**Figure 4:**
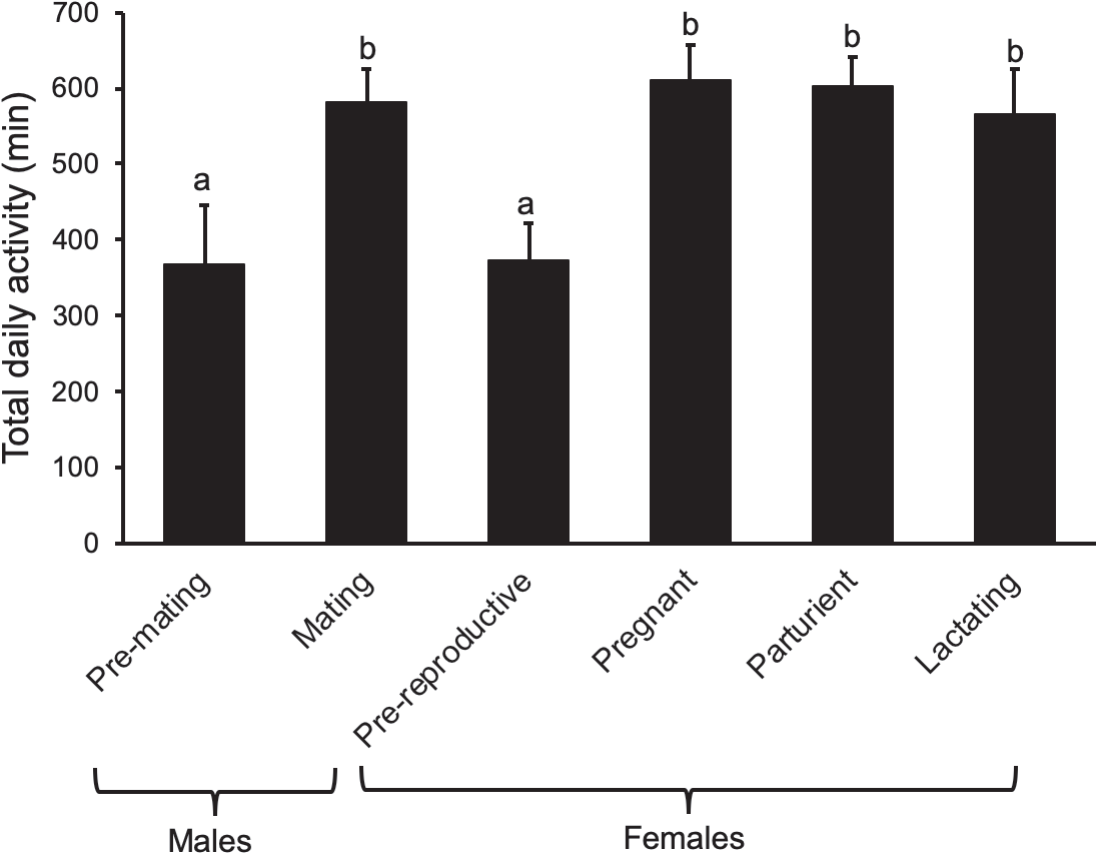
Daily mean activity duration (TAD; minutes per day) is compared among reproductive groups. Significantly different groups are marked with different letters (post hoc Tukey test).

Both reproductive group (*t*_13,697_ = −6.5, *P* < 0.0001, lme) and daily *T*_a min_ (*t*_13,697_ = 3.9, *P* < 0.0001, lme) displayed a significant effect on total daily activity duration (TAD). TAD was greatest for pregnant females, which were active for an average of 610.3 ± 46.9 min per day (*n* = 5, *N* = 108). This was similar to mating males (582.2 ± 43.4 min per day, *n* = 3, *N* = 34; *P* = 0.979, *z* = −0.70, post hoc Tukey test), parturient females (603.0 ± 37.9 min per day, *n* = 4, *N* = 13; *P* = 0.999, *z* = −0.16, post hoc Tukey test) and lactating females (566.7 ± 57.8 min per day, *n* = 3, *N* = 29; *P* = 0.809, *z* = 1.21, post hoc Tukey test; Fig. 4). These activity periods were significantly higher (*P* < 0.001 for these comparisons, post hoc Tukey test) than the TAD of both pre-reproductive females, which foraged on average for 372.9 ± 48.9 min per day (*n* = 8, *N* = 371), and of pre-mating males, which were active for 368.6 ± 78.3 min per day (*n* = 6, *N* = 163; pre-reproductive females and males did not differ; *P* = 0.997, *z* = 0.47, post hoc Tukey test).

## Discussion

We show that *A. flavipes* use a change in activity and adjust physiological mechanisms, such as daily torpor, to meet the varying energetic demands they encounter during their reproductive period. Females expressed at least three distinct torpor patterns which coincided broadly with changes in reproductive state, whereas males expressed two varying torpor patterns before and during the mating period. Activity patterns also changed following conception, as females increased activity and likely food intake, rather than entering torpor to meet energetic requirements.

Pre-reproductive females used torpor extensively during this study, with torpor depth, duration and frequency being significantly higher than in any other group. The frequency of torpor for pre-reproductive females was exceptional for antechinus, with torpor use on 83.0 ± 10.4% of days. Such a high expression of torpor in this species has only been observed in a denuded post-fire environment with low food availability (Matthews *et al*., 2017). However, wild numbats (*Myrmecobius fasciatus*) increase torpor use from 28.6% of nights during autumn up to 90% of nights during winter (Cooper and Withers, 2004) and arid zone dunnarts, *Sminthopsis* spp., use torpor up to 100% of days in winter (Warnecke *et al*., 2008). Pre-reproductive females also had the greatest daily range of *T*_b_ fluctuations, with one of the lowest *T*_b_ values recorded for torpid antechinus (*T*_b min_ 16.6°C, well below previous measurements for the species of >20°C). The *T*_a_ affected both *T*_b_ and activity patterns in this reproductive group, with longer and deeper torpor bouts, and less activity at low *T*_a_, likely reflecting higher thermoregulatory costs and a reduced insect abundance. Further, pre-reproductive females were in general less active in comparison to the other female reproductive groups, which indicates these individuals favour torpor use as a mechanism for dealing with the energetic challenges posed by a low *T*_a_ and food availability during winter. Deep and prolonged torpor use by female dasyurids prior to reproduction and parturition has been interpreted as an energy storing strategy because of the resulting increase in fat deposition, which can later be metabolized during the energetically demanding period of lactation (Geiser and Masters, 1994). An increase in *T*_a_ due to a warming climate may reduce the amount of energy saved and fat deposited during torpor bouts and therefore negatively impact reproductive success.

Pregnant females appeared to only enter torpor during the earlier stages of pregnancy, and on average their torpor bouts were much shorter and shallower than in pre-reproductive females. These shorter torpor bouts were accompanied by an increase in activity duration, likely needed to meet their increasing energetic demands. Torpor use is possible throughout the early stages of pregnancy in most heterothermic marsupials because the young are typically very small (<0.5% BM of mother) and under-developed when born; therefore, they require comparatively little energy and nutrients for growth (Cork, 1991; Geiser and Masters, 1994; Munks and Green, 1995; Körtner *et al*., 2008). While the negative implications of torpor use in relation to slowing and interrupting the growth and development of young are therefore likely to be minimal at least during early pregnancy for these marsupials, they do still decrease torpor use during the course of pregnancy, suggesting some trade-offs. A mismatch between food availability and energy or nutrient availability due to a changing climate and unpredictable conditions may necessitate an increase in the use of torpor during pregnancy, which would delay birth. A delay of birth will result in a shift of the lactation period and therefore reduce the time available for growth and development of young, which likely will cause energy challenges when the next cold season and breeding period arrive. However, some invertebrate populations may increase under certain climate scenarios, which could provide enough energy for antechinus throughout gestation to avoid torpor use.

Energy expenditure increases significantly during lactation, in particular late lactation, for many marsupials (Cork, 1991; Munks and Green, 1995). Parturient and lactating females did not enter torpor at all, and both reproductive states are characterized by a tight control of normothermic *T*_b_. Although the difference in *T*_b range_ between these two groups was not significant, lactating females expressed slightly lower *T*_b min_, which may provide metabolic savings during the rest phase (Levesque *et al*., 2016). Such slight fluctuations of *T*_b_ are unlikely to compromise milk production and the subsequent growth of young, which would be incurred at low very *T*_b_ (Racey and Swift, 1981; Wilde *et al*., 1999). Conversely, the maintenance of a highly stable *T*_b_ as observed during the 5-day parturition period indicates this time is critical for proper development, and even slight reductions in *T*_b_ could have negative implications. While such a reduction in the amplitude of *T*_b_ has been found for other mammals, it often occurs throughout the entire pregnancy period (Scribner *et al*., 1994; Friebe *et al*., 2014; Trethowan *et al*., 2016), rather than being restricted to a few days near parturition. Some domestic animals show a decrease in *T*_b_ immediately prior to parturition (Ewbank, 1969; King *et al*., 1972); however, this was not observed in the current study.

The timing of weaning in *A. flavipes* is closely matched with the arrival of spring and corresponding flushes of arthropods; therefore, any developmental delays due to torpor may lead to a disadvantage, which is crucial especially for semelparous species (Woolley, 1966). Activity patterns in parturient and lactating females were similar to those of pregnant females and in these reproductive groups are likely to be a consequence of increased energy and nutrient supply associated with reproduction, which can no longer be met by relying on torpor. Pregnant, parturient and lactating females therefore meet their energetic demands to a large extent by increasing foraging and thus food intake. Importantly, if antechinus do experience a mismatch in timing of the lactation period and a suitable supply of food due to a changing climate, and if torpor is incompatible with this reproductive state for antechinus, the mother may perish before weaning her young and therefore population recruitment will be significantly hampered. Further, if the mother has to forage more frequently and widely to find sufficient food, this would also increase her exposure to predators. These factors combined could potentially result in local population extinctions.

Torpor displayed by both pre-mating and mating male *A. flavipes* was shorter, shallower and less frequent than for pre-reproductive females. This is in agreement with findings on less pronounced torpor for males than females across a number of dasyurids (Körtner *et al*., 2008; Rojas *et al*., 2014). The observed difference of torpor patterns between the sexes in *A. flavipes* was originally thought to be to a large extent a function of BM, as males were substantially heavier than females (Geiser, 1988), and BM and daily torpor expression are inversely related (Ruf and Geiser, 2015). In our study, however, males were not significantly heavier than females, and therefore the observed thermoregulatory differences may reflect alternate energetic requirements arising from differing reproductive activities (Körtner *et al*., 2010). Interestingly, torpor use was more pronounced and *T*_b_ lower for males before the 2-week mating period. This is in agreement with the finding that pre-mating male *A. flavipes* were least active of all groups, but similar to pre-reproductive females. However, during the mating period males did significantly increase activity, suggesting that perhaps before this period they were also saving energy stores for this more energetically demanding period. During these 2 weeks, males need to not only seek out females, but defend their nest and home ranges from rival males and also have enough energy for their prolonged copulation events that can last up to 12 h (Woolley, 1966). Further, male *A. stuartii* have been shown to display lekking behaviour, with males nesting in large aggregations during the mating period (Lazenby-Cohen and Cockburn, 1998; Cockburn and Lazenby-Cohen, 1992). During the current study, we also observed male *A. flavipes* nesting together during the mating period, suggesting that lekking behaviour may also occur in this antechinus species and the importance of conserving large tree hollows to provide space for these leks.

All males in our study were either confirmed deceased or undetectable approximately 3 weeks after conception occurred in the first of the females. This result is consistent with the known annual die-off which generally occurs within 1 month of breeding (Smith, 1984). Death is usually the result of gastrointestinal haemorrhage, and a compromised immune system which results in bacterial infections and heavy parasite loads (Barker *et al*., 1978). The two male individuals we recovered in this study appeared to have been predated, and while the fate of the remaining four males that disappeared cannot be known with certainty, the lack of a transmitter signal suggests these animals may also have fallen prey to predators that took the animals far from the study site. A heavy parasite burden and ill health in post-mating males would almost certainly make them easy targets for predators. As some parasites will flourish in a warmer climate (Lamarre *et al*., 2018), male antechinus may perish more rapidly, limiting their reproductive potential.

Our study provides further evidence that daily torpor has alternative benefits other than just short-term energy savings for energetically stressed individuals (Nowack *et al*., 2017; Reher *et al*., 2018; Besler and Broders, 2019). Torpor may be employed by many heterothermic endotherms before and early during reproduction to enhance reproductive success by facilitating additional fat storage to be accessed later during pregnancy and lactation (Willis *et al*., 2006; Morrow and Nicol, 2009; Stawski, 2010; Dzal and Brigham, 2013; Klug and Barclay, 2013; McAllan and Geiser, 2014). This may minimize reproductive trade-offs, such as small litter sizes, and improve the chance of survival for mother and young. Therefore, torpor use is a vital aspect of the life history of *A. flavipes* and many other small mammals in relation to reproduction and therefore population recruitment, suggesting that models predicting population changes and conservation management policies need to include such physiological data. Our data are the first to report on antechinus in the wild during their reproductive period, which is a vital initial step in developing an understanding of how these species respond to environmental change during a crucial component of their life history and provide the tools to create such predictive models. Importantly, as many marsupial species, including *A. flavipes*, commence the reproductive period in relation to photoperiod rather than seasonal changes in weather, these species may be at risk of population collapse as a result of a changing climate. As marsupials in Australia are already experiencing significant extinction rates, more studies on the interaction between physiological variables, such as torpor use, and reproduction are needed because reproductive success is critical to population recruitment and therefore is pertinent to the conservation of small mammals.

## References

[ref1] Barker IK, Beveridge I, Bradley AJ, Lee AK (1978) Observations on spontaneous stress-related mortality among males of the dasyurid marsupial *Antechinus stuartii* Macleay. Aust J Zool 26: 435–447.

[ref2] Barclay RMR, Lausen CL, Hollis L (2001) What’s hot and what’s not: defining torpor in free-ranging birds and mammals. Can J Zool 79: 1885–1890.

[ref3] Besler NK, Broders HG (2019) Combinations of reproductive, individual, and weather effects best explain torpor patterns among female little brown bats (*Myotis lucifugus*). Ecol Evol 9: 5158–5171. 31110669 10.1002/ece3.5091 PMC6509385

[ref4] Boyer BB, Barnes BM (1999) Molecular and metabolic aspects of mammalian hibernation: expression of the hibernation phenotype results from the coordinated regulation of multiple physiological and molecular events during preparation for and entry into torpor. BioScience 49: 713–724.

[ref5] Bradley AJ (2003) Stress, hormones and mortality in small carnivorous marsupials In Jones M, Dickman C, Archer M, eds, Predators with Pouches – The Biology of Carnivorous Marsupials. CSIRO Publishing, Victoria, pp. 255–267.

[ref6] Cockburn A, Lazenby-Cohen KA (1992) Use of nest trees by *Antechinus stuartii*, a semelparous lekking marsupial. J Zool 226: 657–680.

[ref7] Cooper CE, Withers PC (2004) Patterns of body temperature variation and torpor in the numbat, *Myrmecobius fasciatus* (Marsupialia: Myrmecobiidae). J Therm Biol 29: 277–284.

[ref8] Cork SJ (1991) Meeting the energy requirements for lactation in a macropodid marsupial: current nutrition versus stored body reserves. J Zool 225: 567–576.

[ref9] Dzal YA, Brigham RM (2013) The tradeoff between torpor use and reproduction in little brown bats (*Myotis lucifugus*). J Comp Physiol B 183: 279–288. 22972361 10.1007/s00360-012-0705-4

[ref10] Ewbank R (1969) The fall in rectal temperature seen before parturition in sheep. Reproduction 19: 569–571. 10.1530/jrf.0.0190569 5809477

[ref11] Fisher DO, Dickman CR, Jones ME, Blomberg SP (2013) Sperm competition drives the evolution of suicidal reproduction in mammals. Proc Natl Acad Sci USA 110: 17910–17914. 24101455 10.1073/pnas.1310691110 PMC3816400

[ref12] Friebe A, Evans AL, Arnemo JM, Blanc S, Brunberg S, Fleissner G, Swenson JE, Zedrosser A (2014) Factors affecting date of implantation, parturition, and den entry estimated from activity and body temperature in free-ranging brown bears. PLoS One 9: e101410. 24988486 10.1371/journal.pone.0101410 PMC4079694

[ref13] Geiser F (1988) Daily torpor and thermoregulation in antechinus (Marsupialia): influence of body mass, season, development, reproduction, and sex. Oecologia 77: 395–399. 28311957 10.1007/BF00378050

[ref14] Geiser F, Masters P (1994) Torpor in relation to reproduction in the mulgara, *Dasycercus cristicauda* (Dasyuridae: Marsupialia). J Thermal Biol 19: 33–40.

[ref15] Geiser F, Turbill C (2009) Hibernation and daily torpor minimize mammalian extinctions. Naturwissenschaften 96: 1235–1240. 19578825 10.1007/s00114-009-0583-0

[ref16] Geiser F, Holloway J, Körtner G, Maddocks TA, Turbill C, Brigham RM (2000) Do patterns of torpor differ between free-ranging and captive mammals and birds? In Heldmaier G, Klingenspor M, eds, Life in the Cold. 11th International Hibernation Symposium. Berlin Heidelberg. Springer, New York, pp. 95–102.

[ref17] Hanna E, Cardillo M (2014) Clarifying the relationship between torpor and anthropogenic extinction risk in mammals. J Zool 293: 211–217.

[ref18] Johnson C (2006) Australia’s Mammal Extinctions: a 50,000-Year History. Cambridge University Press, Cambridge.

[ref19] King GJ, Willoughby RA, Hacker RR (1972) Fluctuations in rectal temperature of swine at parturition. Can Vet J 13: 72–74. 5016929 PMC1695641

[ref20] Klug BJ, Barclay RMR (2013) Thermoregulation during reproduction in the solitary, foliage-roosting hoary bat (*Lasiurus cinereus)*. J Mammal 94: 477–487.

[ref21] Körtner G, Geiser F (1998) Ecology of natural hibernation in the marsupial mountain pygmy-possum (*Burramys parvus*). Oecologia 113: 170–178. 28308194 10.1007/s004420050365

[ref22] Körtner G, Pavey CR, Geiser F (2008) Thermal biology, torpor and activity in free-living mulgaras in arid zone Australia during the winter reproductive season. Physiol Biochem Zool 81: 442–451. 18507533 10.1086/589545

[ref23] Körtner G, Rojas AD, Geiser F (2010) Thermal biology, torpor use and activity patterns of a small diurnal marsupial from a tropical desert: sexual differences. J Comp Physiol B 180: 869–876. 20217093 10.1007/s00360-010-0459-9

[ref24] Lamarre V, Legagneux P, Franke A, Casajus N, Currie DC, Berteaux D, Bêty J (2018) Precipitation and ectoparasitism reduce reproductive success in an arctic-nesting top-predator. Sci Rep 8: 8530. 29867211 10.1038/s41598-018-26131-y PMC5986809

[ref25] Lazenby-Cohen KA, Cockburn A (1998) Lek promiscuity in a semelparous mammal) *Antechinus stuartii* (Marsupialia: Dasyuridae)? Behav Ecol Sociobiol 22: 195–202.

[ref26] Lee AK, Cockburn A (1985) Evolutionary Ecology of Marsupials. Cambridge University Press, Cambridge.

[ref27] Levesque DL, Nowack J, Stawski C (2016) Modelling mammalian energetics: the heterothermy problem. *Clim Change Resp* 3: 1–11.

[ref28] Matthews JK, Stawski C, Körtner G, Parker CA, Geiser F (2017) Torpor and basking after a severe wildfire: mammalian survival strategies in a scorched landscape. J Comp Physiol B 187: 385–393. 27734149 10.1007/s00360-016-1039-4

[ref29] McAllan BM, Dickman CR (1986) The role of photoperiod in the timing of reproduction in the dasyurid marsupial *Antechinus stuartii*. Oecologia 68: 259–264. 28310137 10.1007/BF00384797

[ref30] McAllan BM, Geiser F (2006) Photoperiod and the timing of reproduction in *Antechinus flavipes* (Dasyuridae: Marsupialia). Mamm Biol 71: 129–138.

[ref31] McAllan BM, Geiser F (2014) Torpor during reproduction in mammals and birds: dealing with an energetic conundrum. Int Comp Biol 54: 516–532. 10.1093/icb/icu093 24973362

[ref32] Morrow G, Nicol SC (2009) Cool sex? Hibernation and reproduction overlap in the echidna. PLoS ONE 4: e6070. 19562080 10.1371/journal.pone.0006070 PMC2699653

[ref33] Munks SA, Green B (1995) Energy allocation for reproduction in a marsupial arboreal folivore, the common ringtail possum (*Pseudocheirus peregrinus*). Oecologia 101: 94–104. 28306981 10.1007/BF00328905

[ref34] Naylor R, Richardson S, McAllan B (2008) Boom and bust: a review of the physiology of the marsupial genus *Antechinus*. J Comp Physiol B 178: 545–562. 18210128 10.1007/s00360-007-0250-8

[ref35] Nowack J, Stawski C, Geiser F (2017) More functions of torpor and their roles in a changing world. J Comp Physiol B 187: 889–897. 28432393 10.1007/s00360-017-1100-y PMC5486538

[ref36] Racey PA, Swift SM (1981) Variations in gestation length in a colony of pipistrelle bats (*Pipistrellus pipistrellus*) from year to year. J Reprod Fertil 61: 123–129. 7452610 10.1530/jrf.0.0610123

[ref37] Reher S, Ehlers J, Rabarison H, Dausmann KH (2018) Short and hyperthermic torpor responses in the Malagasy bat *Macronycteris commersoni* reveal a broader hypometabolic scope in heterotherms. J Comp Physiol B 188: 1015–1027. 30121696 10.1007/s00360-018-1171-4

[ref38] Rojas AD, Körtner G, Geiser F (2010) Do implanted transmitters affect maximum running speed of two small marsupials? J Mammal 91: 1360–1364.

[ref39] Rojas AD, Körtner G, Geiser F (2014) Torpor in free-ranging antechinus: does it increase fitness? Naturwissenschaften 101: 105–114. 24441710 10.1007/s00114-013-1136-0

[ref40] Ruf T, Geiser F (2015) Daily torpor and hibernation in birds and mammals. Biol Rev 90: 891–926. 25123049 10.1111/brv.12137 PMC4351926

[ref41] Scribner SJ, Wynne-Edwards KE (1994) Disruption of body temperature and behavior rhythms during reproduction in dwarf hamsters (*Phodopus*). Physiol Behav 55: 361–369. 8153179 10.1016/0031-9384(94)90147-3

[ref42] Selwood L, Woolley PA (1991) A timetable of embryonic development, and ovarian and uterine changes during pregnancy, in the stripe-faced dunnart, *Sminthopsis macroura* (Marsupialia: Dasyuridae). J Reprod Fertil 91: 213–227. 1995850 10.1530/jrf.0.0910213

[ref43] Smith G (1984) The biology of the yellow-footed antechinus, *Antechinus flavipes* (Marsupialia: Dasyuridae), in a swamp forest on Kinaba Island, Cooloola Queensland. Wildl Res 11: 465–480.

[ref44] Stawski C (2010) Torpor during the reproductive season in a free-ranging subtropical bat, *Nyctophilus bifax*. J Thermal Biol 35: 245–249.

[ref45] Stawski C, Rojas AD (2016) Thermal physiology of a reproductive female marsupial, *Antechinus flavipes*. Mamm Res 61: 417–421.

[ref46] Trethowan PD, Hart T, Loveridge AJ, Haw A, Fuller A, Macdonald DW (2016) Improved homeothermy and hypothermia in African lions during gestation. Biol Lett 12: 20160645. 27881765 10.1098/rsbl.2016.0645 PMC5134044

[ref47] Warnecke L, Turner JM, Geiser F (2008) Torpor and basking in a small arid zone marsupial. Naturwissenschaften 95: 73–78. 17684718 10.1007/s00114-007-0293-4

[ref48] Wilde CJ, Knight CH, Racey PA (1999) Influence of torpor on milk protein composition and secretion in lactating bats. J Exp Zool 284: 35–41. 10368932 10.1002/(sici)1097-010x(19990615)284:1<35::aid-jez6>3.0.co;2-z

[ref49] Willis CKR (2007) An energy-based body temperature threshold between torpor and normothermia for small mammals. Physiol Biochem Zool 80: 643–651. 17910000 10.1086/521085

[ref50] Willis CKR, Brigham RM, Geiser F (2006) Deep, prolonged torpor by pregnant, free-ranging bats. Naturwissenschaften 93: 80–83. 16456644 10.1007/s00114-005-0063-0

[ref51] Withers PC, Cooper CE, Maloney SK, Bozinovic F, Cruz-Neto AP (2016) Ecological and Environmental Physiology of Mammals. Oxford University Press, Oxford.

[ref52] Woinarski JCZ, Burbidge AA, Harrison PL (2015) Ongoing unravelling of a continental fauna: decline and extinction of Australian mammals since European settlement. Proc Natl Acad Sci USA 112: 4531–4540. 25675493 10.1073/pnas.1417301112 PMC4403217

[ref53] Woolley P (1966) Reproduction in *Antechinus* spp. and other dasyurid marsupials. Symp Zool Soc London 15: 281–294.

